# Community Engagement to explore Surrogate Decision-Making for HIV+ African Americans: a pilot study

**DOI:** 10.18103/mra.v13i11.6823

**Published:** 2025-11

**Authors:** Mei Ching Lee, Randy Woods, Mian Bazle Hossain, Ila Mulasi, Renard Murray, Carla S Alexander, members of the Community Advisory Panel (CAP) Yvonne Henley, Frank Oldham, Steven Eveland, Robyn Palmiero LCSW

**Affiliations:** 1 University of Maryland Baltimore School of Nursing; 2 Sisters Together and Reaching, Inc. (CBO); 3 Morgan State University School of Public Health - Biostatistics; 4 University of Maryland Baltimore School of Medicine – Palliative Care; 5 University of Maryland Baltimore School of Medicine – Institute of Human Virology; 6 DECIDE Study Community Advisory Panel

**Keywords:** Advance Care Planning, African American, HIV/AIDS, Surrogate Decision-Maker, Community Engagement, chronic serious illness, Training Support, Trust - Trustworthiness, syndemics, anthropology

## Abstract

**Background:**

Patient-centered communication and trusting relationships are central to effective clinical management for anyone living with chronic illness. For those able to access and continue effective medications, aging with the human immunodeficiency virus (HIV) is now like aging with any chronic illness. Advance Care Planning (ACP) has been a recognized strategy to improve health outcomes for individuals with serious illnesses. Despite its importance, African Americans living with HIV (AAHIV) often experience barriers to ACP, including having lived in a syndemic environment where low health literacy and historical mistrust of the healthcare system are inherent. Our clinically translational pilot study aimed to evaluate the feasibility and acceptability of discussing surrogate decision-making and ACP within an urban syndemic using a community-engaged research approach to improve individual outcomes.

**Methods:**

This was a one-year translational research pilot, to tryout a newfound academic-community research team formed during COVID-19 in one eastern urban setting. We used Community Engagement in Research (CEnR) and mixed methods to evaluate the impact of community input on Focus Group activities. Participants were recruited through local HIV/AIDS service organizations. A 19-item survey assessed ACP awareness and behaviors (n=75) and focus group discussion (n=34) explored barriers and facilitators of surrogate decision-making prompted by a community-generated scenario. Data analysis included descriptive statistics for the survey and thematic analysis for focus group discussions.

**Results:**

DECIDE survey results indicated that after the COVID pandemic, 45% of participants had never heard of ACP, and, of those with prior knowledge, 73% had discussed their treatment preferences, only 37% had created an ACP document. Approximately 49% of participants had named a health proxy, although only 45% of those individuals had discussed their values with the proxy. Use of a community-constructed scenario led to rapid assembly and valuable discourse among focus group participants despite the discussion being about dying. Focus group findings revealed a strong desire for more opportunities to rehearse ACP conversations and a persisting mistrust of healthcare systems. Participants expressed the need for continued, community-based educational efforts to address these issues.

**Conclusion:**

The DECIDE translational pilot endorses the literature that community engagement is feasible, acceptable and effective in syndemic settings. Use of a community-generated scenario rapidly engaged participants in meaningful talk about surrogate decision-making among African Americans living with HIV. CEnR accelerated data collection about a potentially avoided topic for participants living in syndemic settings. Future research should address systemic barriers, trust-building, and community engagement in sustainable educational activities for improving community health practices.

## Introduction

The United States (US) population is aging with 25% of persons expected to be over 65 years of age in 2060^1^. This increase in age and associated comorbidities requires a shift from episodic treatment of acute illness toward greater continuity of care for chronic conditions requiring both clinical and social care^2^. Approximately 1.2 million people in the US and dependent areas are living with the chronic condition of human immunodeficiency virus (HIV) reflecting the ongoing worldwide pandemic^3^. Currently, over 600,000 HIV+ persons are over 50 years of age in the US with an equivalent life expectancy of anyone suffering with multiple comorbidities^4^. Annual diagnoses reflect male-to-male sexual contact accounting for 71% of the HIV infection with 43% of those being African American^5^. The highest HIV transmission rates continue to occur in syndemic settings where people are often socially isolated and must contend with overlapping social determinants of health^6^.

In 2022, the World Health Organization (WHO) published consolidated guidelines as a public health response to HIV, viral hepatitis, and sexually transmitted infections for five key populations and their networks to highlight the critical role of addressing structural barriers, such as syndemics, when striving to minimize the impact of these infections^7^.Palliative care, a coordinated and practical approach to care and support across the lifespan, is now recognized as an essential component of the HIV care continuum^8^. Both rural and urban areas have been labeled as syndemic environments where poverty, substance abuse, childhood trauma and/or personal responses, e.g., hypervigilance seen with post-traumatic stress disorder (PTSD), impede the individual and the community from being future-focused^9–11^. Because key populations have experience stigma and discrimination, or felt uncomfortable, in tertiary hospitals, decentralization of services and the use of peer navigators have been recommended to increase acceptability and accessibility of service delivery. Person-centered communication and trusting relationships are central to effective clinical management for persons living with HIV illness^5. 9–10, 12–14^.

### REVIEW OF EVIDENCE

Advance Care Planning (ACP) is a recognized palliative strategy to improve health outcomes for individuals with serious illness^15^. It has been associated with improved patient outcomes, including increased satisfaction with care, better alignment of care with patient values, and reduced hospital admissions when a person is nearing the end-of-life^16^. ACP is the process of planning for future medical decisions if a person becomes unable to communicate his/her/their wishes^17^. Families and friends, if asked by the index patient, can participate in discussions about treatment preferences, designating and educating a surrogate decision-maker, and documenting those preferences in an advance directive. A recent randomized-controlled trial demonstrated that factors extrinsic to specific ACP interventions influence how prepared spokespersons feel to act as spokespersons. Occurrence of post-intervention ACP conversations did not influence perceived preparedness; however, spokespersons who used an ACP decision aid reported more conversations^18^. Examples of such learning aids include PREPARE for your Care^16^; FACE (FAmily-CEntered)^19^; and SPIRIT^20^.

Without having explicit conversations about ACP, surrogate, or proxy, decision-makers identified in the hospital are unlikely to truly represent the wishes of the individual^21^. A pre-COVID systematic review of ACP in the HIV population showed that the prevalence was variable with 36%–54% having had any end-of-life communication and only 8%–47% having created advance directives^22^. Individual studies documented that 30 to 90% of persons living with HIV lacked trust for identifying a surrogate decision-maker^22^. Later, in 2021, 100 men living with HIV during the COVID-19 pandemic documented that having an advance directive was associated with older age, higher education, living with other people, never having had an AIDS diagnosis, and undetectable viral load (*p* < 0.05)^23^. Despite the known benefits, ACP remains underutilized among African Americans with low health literacy and historical mistrust of the healthcare system^24^, particularly those African Americans living with HIV (AAHIV)^25^. The HIV pandemic has influenced clinical care delivery and research in multiple areas as itemized by Johnson and including virology advances allowing for a scientifically rapid creation of SARS vaccine^26^.

### CONTRIBUTION TO THE FIELD

In this paper, we describe a translational pilot study using collective (US) action (UK) research to inform future approaches for implementing ACP, a complex intervention, for those living with chronic illness using HIV disease as a model. The DECIDE (Discover by Engaging Community in Decision-Making for Empowerment) pilot used community-engagement research to evaluate the feasibility and acceptability of accelerating ACP discussions including identification of anyone as a proxy, or surrogate decision-maker, within one syndemic setting. We aimed to identify factors that impact engagement in the ACP process and surrogate decision-making in a syndemic population with a focus on participatory discussion for individual empowerment. Preparing surrogates for participating in hospital-based ACP is a critical aspect that has been neglected^16^. DECIDE established a partnership among academic institutions and local community organizations, leveraging community knowledge and trust to facilitate effective dialog about ACP. Our earlier studies about integrating care approaches with existing healthcare delivery required an HIV Advisory Panel that was modified for the DECIDE pilot study^27–28^. The field of ACP continues to evolve. A framework for evaluating 5 areas of outcomes: Process; Action; Quality of Care; Health; and Health Systems was introduced in 2024 for organization of future research^29^.

## Methods

### STUDY DESIGN

The DECIDE study was a 12-month, mixed-methods translational pilot designed to assess the feasibility and acceptability of community-based ACP interventions for AAHIV about preparation for proxy decision-making. The study was conducted in collaboration with two schools in one University (Schools of Nursing and Medicine), Sisters Together and Reaching, Inc. (STAR), a faith-based non-profit organization, and another University School of Public Health (Social Biostatistics) with a Community Advisory Panel of volunteers who have participated in other studies as well as individuals interested in ACP. The study was approved by the Institutional Review Board (IRB) prior to interaction with participants.

### STUDY POPULATION

Participants were recruited through flyers distributed by local HIV/AIDS service organizations and outreach to African Americans living with HIV. The inclusion criteria were adults aged 18 and older, residing in the Baltimore area, and living with HIV disease. A total of 75 individuals completed the survey, and 34 individuals participated in focus group discussions.

### DATA COLLECTION

The 19-item survey was developed to assess participants’ awareness of ACP, their past experiences with ACP, and their behaviors related to surrogate decision-making. The survey included nine quantitative items on ACP behaviors (e.g., completion of ACP documents, discussions about treatment preferences), eight opinion-based items regarding the importance of ACP and surrogate decision-making, and two demographic questions (age, gender). Surveys were distributed during community outreach events, and participants were given a small incentive for their time. Descriptive statistics were used to summarize responses. Four 90-minute focus groups were conducted (two in-person and two virtual) with a total of 34 participants, 22 males and 12 females. Focus groups were moderated by a team of nursing and medical faculty, with co-leadership from community advisory panel (CAP) members. A semi-structured moderator guide was used, with open-ended questions designed to explore participants’ attitudes toward ACP and surrogate decision-making, specifically their experiences with naming and preparing a proxy.

### STATISTCAL ANALYSIS

Quantitative data analysis was conducted using descriptive statistics (frequencies, percentages, and means) to summarize participants’ ACP behaviors and demographic characteristics. For qualitative data, all focus groups were audio recorded and transcribed by a professional transcriber. Two study team members conducted thematic analyses to improve the rigor and transparency ^30^. The analysis focused on identifying recurring themes related to ACP, surrogate decision-making, and barriers to engagement.

## Results

A total of 75 participants completed the DECIDE survey. Demographic characteristics included 61% females, 39% males, and an age range from 18 to 65 years. The survey results revealed that 45% of participants had never heard of ACP, and 37% had completed an ACP document. In addition, 73% of participants had discussed their treatment preferences with someone, though only 49% had named a health proxy. Of the 49% who had named a health proxy, 45% had discussed their treatment values and preferences with that proxy. However, 21% of participants expressed concerns that their proxy would not be able to represent their wishes accurately. Furthermore, 44% of participants had served as a healthcare proxy for someone else, and 76% agreed or strongly agreed that education about how to talk with a proxy would be helpful (Table 1).

Focus Group discussions revealed several key themes. “Trust” was raised as a major factor to be improved upon. There were concerns about mistrust in the healthcare system, particularly among those who had experienced discrimination in medical settings. Participants felt strongly about “trust” as a key consideration for having an honest conversation about their wishes (all groups). The “trust” was related to their relationships to the surrogates (group 3,4), the surrogate’s ability in make decisions (group 2, 3, 4), carrying out their wishes (group 3, 4), and the ability to control emotion in chaos situation (group 2, 3, 4). Participants also discussed “trust” with the health providers and expressed “not having support, especially mentally;” “primary clinician did not mention it (the prognosis);” and “not explaining (the health condition)” (group 2). Participants cited societal stigma around HIV as a barrier to ACP conversations (all groups).

Participants expressed a strong desire for ongoing opportunities to practice ACP conversations. Many indicated that ACP should be a process rather than a one-time event, with ample time for rehearsal and preparation. Participants stated the discussion about their wishes was a “hard topic” and “needing to have an opportunity for the discussion.” (group 2, 4). One group pointed out that not all surrogates will “accept (or follow) my wishes” (group 4).

Participants reported that they often lacked the knowledge to effectively communicate their values and preferences to a healthcare proxy (group 2, 4). Couple participants reported “I have never heard of this information and have no clue (of what surrogate is)” (group 4). “I know nothing, its hard” (group 3), “someone needs to explain the medical terms to me” (group 3), “I need someone to help process the thoughts” (group 2). Preparing a “stand-in” through education about health literacy, including how to express one’s wishes, was seen as crucial.

All groups agreed that culturally relevant education was essential to promoting ACP. Content of this educational experience should allow time for learning to express one’s own wishes for future decision-making as well as opportunities to practice ACP conversations before being expected to participate in a family meeting in a hospital setting. Being able to appoint and trust that a “stand-in” could effectively convey one’s wishes would require education for the “stand-in” as well. Avoiding direct references to death and dying was voiced as these words can recall past traumatic experience and invoke fear that could derail the conversation.

## Discussion

In the 1960s, there was public debate about how medical decision-making was accomplished inside the hospital, especially near the end of life. Over the next decade health care advance directives were promoted as the primary legal tool to communicate formally one’s health care wishes^31^. For forty years the controversy of whose wishes are being respected in healthcare decision-making has continued to prompt debate. Although legislation was introduced in the mid-1970’s, advance care planning has not been fully implemented in the US^17^.In our pilot, ACP provides the patient with a “voice” to reduce stress for the “stand-in” by being prepared for what will be asked and what the patient wants. It is in this context that we recognize ACP a ‘cross-cutting issue’ for healthcare decision-making in persons with any serious illness.

The DECIDE pilot is a clinical translational pilot addressing individual factors blocking universal adoption of the ‘cross cutting issue’ of advance care planning. ACP is a complex intervention and, as such, has been challenging to fully study. Within the HIV population, there are several distinct communities that have not been able to complete the full ACP process. We have recognized that a syndemic environment along with ingrained beliefs and practices has resulted in members being partially isolated from the mainstream. We used community-based, culturally tailored methods to engage African American adults living with HIV in one urban syndemic area to discuss ACP topics. Community engagement is well described in the social sciences with the original theory and components described in a publication by Edward Freeman^32^. Rubin and Babbie expanded the concept of stakeholders as “those with vested interest^14^ in processes and outcomes. This interpretation of “stakeholders” underscores the importance of recognizing both direct, and/or indirect, concerns in successful integration of new practices^33–34^.

### DECIDE POST-COVID DATA COLLECTION

Our quantitative data was collected by simple survey. Although a significant portion of participants had heard of ACP or discussed treatment preferences, few had completed formal ACP documents nor named a surrogate. This is consistent with previous research on ACP disparities among African Americans^12,19,34–35^. Focus Group Participants were easily engaged in ACP discussions, despite environmental and/or personal barriers, using a community-generated scenario to prompt conversation. Despite well-demonstrated benefits of ACP in the literature, barriers such as low health literacy, mistrust of healthcare systems, and societal stigma around HIV have hindered widespread adoption of ACP. Staff members observed differences in willingness to participate in discussion by age-group. Older focus group members appeared to retain emotional beliefs of earlier lived HIV experience resulting in concerns about privacy and protections not voiced by younger participants. From our prior ACP-related outreach, poignant individual responses about death and dying might derail family dialogue on ACP^12^.

### PERSISTING RACIAL AND SGM STIGMA

Forty-four years after the original HIV diagnoses and improved antiretroviral management, we were surprised by the intensity observed in focus group findings that mistrust in personal relationships with providers and healthcare systems continues. Focus Group Participants had anticipated, or personally experienced, discrimination and this was a key factor contributing to the reluctance to engage in ACP discussions. This aligns with literature suggesting that historical and ongoing racial, sexual and gender minority (SGM) discrimination in healthcare delivery continues to inhibit engagement in end-of-life care planning ^36^. The notion of “surrogate will not follow my wishes” is disconcerting but was not unique in this population, reflecting cultural fears in multiple sub-populations discussing ACP^12^. We did not screen for behavioral health diagnoses, such as post-traumatic stress disorder (PTSD), known to result in signs such as hypervigilance and blaming. PTSD is higher in the HIV population (32.7%) but varies by location and methods of documentation used^37^ This and other behavioral health issues may enhance the intensity seen in Focus Group participants living longer with an HIV diagnosis.

### NEW TARGETS

Despite the overlaid complexity of chronic illness and a syndemic context, “trust building” remains a key target for integrating ACP with disease management, a ‘cross-cutting concept’ for achieving optimal health outcomes, for persons with chronic illness living in syndemic settings. AAHIV adults have requested opportunities to rehearse ACP conversations and for education that is both culturally relevant and sensitive to their lived experiences. Diagnostic instruments to evaluate mental health conditions among participants would improve any HIV study to evaluate the impact of anxiety, depression, PTSD and other behavioral conditions.

The participants in the DECIDE pilot expressed a strong desire for more opportunities to rehearse ACP conversations and for education that is both culturally relevant and sensitive to their lived experiences. This suggests that ACP interventions must be tailored to the unique needs of specific populations. Community stakeholders can vocalize concerns of separate sub-populations within the HIV/AIDS community. They requested time to rehearse responses before having to act as a “stand-in”, or proxy, for someone else. Additional Translational Research is needed to demonstrate and refine community-based learning efforts that will empower individuals to trust and communicate more clearly with health professionals about in-hospital medical decision-making.

### LIMITATIONS

This was a pilot to assure that all team members were prepared for participation in a full study application and to identify any areas that might need to be addressed in a contractual agreement among institutions. We focused upon creating a workable model for a community-academic partnership in research with broader communication issues than studies being conducted within one institution. The study was conceived and submitted during the SARS Pandemic when communication was limited to online discussions. These factors inhibited face-to-face communication among study team members, and we felt that this may have missed opportunities to improve collaboration. While African Americans are not the only sub-population living with HIV disease, those living in syndemic areas have exhibited more difficulty with use of advance care planning as intended in society in general. A 2024 narrative review of the ACP field describes remaining challenges for uniform implementation^38^.

## Conclusion

Despite 40 years of HIV care delivery, AAHIV in one syndemic continue to cite societal stigma around HIV and feel discrimination related to race, sexual behavior and gender. The DECIDE pilot, a clinical translational study using CEnR, has documented that action research is possible and preferred by African Americans living with HIV disease in one syndemic setting where lack of trust and lower health literacy continue to inhibit access to healthcare. Advance care planning known to improve health outcomes for individuals with serious illness, can be discussed and embraced by empowering community members to participate in adapting and facilitating the experience for local use.

Community-engaged educational activities are essential to highlighting the complex beliefs and fears among individuals and cultural groups (stakeholders) aging with chronic illness. Further study is needed to assure that the voice of persons aging in syndemic communities is not disregarded in improving local health delivery.

## Figures and Tables

**Table 1.** Demographic characteristics and ACP* survey results

Characteristics/Survey Responses	Percentage (%)

**Gender**	
Female	61%
Male	39%
**Age range**	18–65 years
**Familiarity with ACP**	
Never heard of ACP	45%
Completed an ACP document	37%
Discussion of treatment preferences	73%
Named a health proxy	49%
**Discussed** treatment values and preferences with proxy	45%
**Concern** that proxy won’t represent wishes accurately	21%
**Served as a healthcare proxy** for someone else	44%
Agreement that education on talking with a proxy would be helpful	76%

* ACP= Advance care planning
